# L290P/V mutations increase ERK3’s cytoplasmic localization and migration/invasion-promoting capability in cancer cells

**DOI:** 10.1038/s41598-017-15135-9

**Published:** 2017-11-03

**Authors:** Hadel Alsaran, Lobna Elkhadragy, Astha Shakya, Weiwen Long

**Affiliations:** Department of Biochemistry and Molecular Biology, Boonshoft School of Medicine, Wright State University, Dayton Ohio, USA

## Abstract

Protein kinases are frequently mutated in human cancers, which leads to altered signaling pathways and contributes to tumor growth and progression. ERK3 is an atypical mitogen-activated protein kinase (MAPK) containing an S-E-G activation motif rather than the conserved T-X-Y motif in conventional MAPKs such as ERK1/2. Recent studies have revealed important roles for ERK3 in cancers. ERK3 promotes cancer cell migration/invasion and tumor metastasis, and its expression is upregulated in multiple cancers. Little is known, however, regarding ERK3 mutations in cancers. In the present study, we functionally and mechanistically characterized ERK3 L290P/V mutations, which are located within ERK3’s kinase domain, and are shown to exist in several cancers including lung cancer and colon cancer. We found that in comparison with wild type ERK3, both L290P and L290V mutants have greatly increased activity in promoting cancer cell migration and invasion, but have little impact on ERK3’s role in cell proliferation. Mechanistically, while they have no clear effect on kinase activity, L290P/V mutations enhance ERK3’s cytoplasmic localization by increasing the interaction with the nuclear export factor CRM1. Our findings suggest that L290P/V mutations of ERK3 may confer increased invasiveness to cancers.

## Introduction

Extracellular signal-regulated kinase 3 (ERK3), also known as MAPK6, is an atypical mitogen-activated protein kinase (MAPK). In contrast to conventional MAPKs (such as ERK1/2) that have dual phosphorylation sites in their conserved T-X-Y activation motif, ERK3 has a single phosphorylation site (S189) in its S-E-G activation motif^1,2^. ERK3 protein shuttles between the nucleus and cytoplasm^3^ and was recently also found to localize at the leading edge of the plasma membrane^4^. Nuclear export of ERK3 is mediated by the exportin protein chromosomal maintenance 1 (CRM1)^3^. In addition, ERK3 was shown to interact with and transport MAPK-activated protein kinase-5 (MK5) from the nucleus to the cytoplasm^5,6^.

Recent studies have revealed important roles for ERK3 in cancers. ERK3 stimulates lung cancer cell invasiveness both *in vitro* and *in vivo* by phosphorylating steroid receptor coactivator 3 (SRC3) oncoprotein and upregulating SRC3-mediated matrix metalloproteinase (MMP) gene expression^7^. In addition, ERK3 regulates cell morphology and promotes breast cancer cell migration^4^. Currently, little is known about the molecular mechanisms underlying ERK3’s motility-promoting role. Furthermore, ERK3 confers lung cancer cells resistance to topoisomerase-2 inhibitors by enhancing the DNA damage repair activity of tyrosyl DNA phosphodiesterase 2 (TDP2)^8^. In line with its important roles in cancer cell migration, invasion and DNA damage response, ERK3 is upregulated in multiple cancers, including non-small cell lung cancer^7^, gastric cancer^9^ and oral squamous cell carcinoma^10^. Mechanistically, ERK3 expression level in cancer cells was shown to be upregulated by BRAF (through increasing ERK3 mRNA)^11^, BMI1 (by suppressing let-7i which targets ERK3 mRNA)^12^ and USP20 (by deubiquitinating and stabilizing ERK3 protein)^13^.

Protein kinases are frequently altered in cancers and are critical players in cancer initiation and progression. Protein kinases can be altered by multiple mechanisms, such as gene copy number changes (gain or loss), mutations (including deletions, chromosomal translocation and point mutations) and epigenetic changes in gene promoters^14,15^. Interestingly, ERK3 mutations were detected in multiple cancers in studies of human genome wide profiling cancer mutations^2,16–18^ and were shown in databases such as COSMIC (Catalogue Of Somatic Mutations In Cancer) and VarSome (the Human Genomic Variant Search Engine). Of important note are the mutations on L290 (L290P and L290V) within the kinase domain of ERK3. Both L290P and L290V mutations were detected in multiple cancers, including lung cancer, skin cancer and colon cancer, albeit at a relatively low frequency (around 1–2%)^2,16–18^. However, the impact of L290P/V mutations on ERK3’s kinase activity and cellular functions remains to be investigated.

In the present study, we generated ERK3 L290P/V mutants and investigated their impact on ERK3 kinase activity and cellular functions. We found that both L290P and L290V mutants greatly promote cancer cell migration and invasion, even significantly more than wild type ERK3, but have little impact on cell proliferation. Mechanistically, while they have no clear effect on kinase activity, L290P/V mutations enhance ERK3’s cytoplasmic localization by increasing the interaction with CRM1. Our findings suggest that L290P/V mutations in ERK3 may confer increased invasiveness to tumors.

## Results

### Both L290P and L290V mutations increase ERK3’s capability of promoting cancer cell migration and invasion

**Figure 1:**
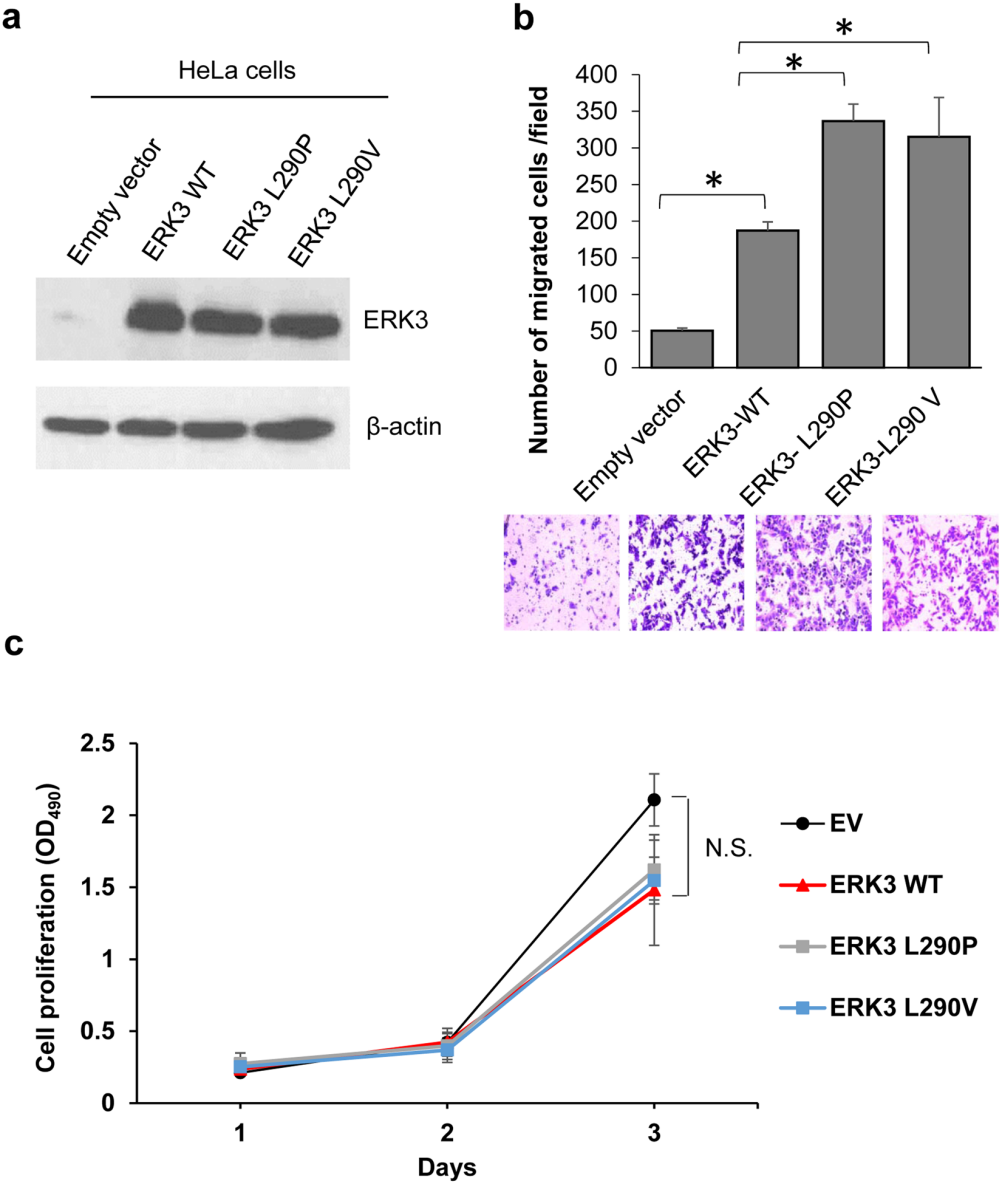
Both ERK3 L290P and ERK3 L290V mutants have increased ability to promote HeLa cell migration as compared to wild type ERK3. (**a**) Western blot analysis of ERK3 and ERK3 mutants’ expression in HeLa cells transfected with either a pSG5 empty vector, wild type ERK3 (ERK3 WT), or each ERK3 L290 mutant as indicated. β-actin was used as a loading control. (**b**) The effect of L290P/V mutations on ERK3’s role in HeLa cell migration. HeLa cells were transfected with each different pSG5 plasmid as indicated. Two days post-transfection, cell migration was analyzed using a two-chamber transwell system. Migrated cells were stained with crystal violet, photographed and counted under a microscope at 50X magnifications. Quantitative results are presented as the number of migrated cells per field. Values in the bar graph represent mean ± S.D. of 3 separate experiments. *P < 0.05 by Student’s *t*-test. Representative images are shown at the bottom. (**c**) The effect of L290P/V mutations on ERK3’s role in HeLa cell proliferation. HeLa cells were transfected with the following plasmids: empty vector (EV), ERK3 WT, ERK3 L290P or ERK3 L290V. Cell growth at different time points (days) was determined by MTS proliferation assay and expressed as OD_490_. Values represent mean ± S.D. of 3 separate experiments. Statistical analysis was conducted by student’s *t-*test. N.S.: no significance.

ERK3 promotes cancer cell migration and invasion^4,7^. We therefore determined the effects of L290P/V mutations on ERK3’s functions in cancer cell migration and invasion. This was first tested in HeLa cells by transiently transfecting HA-tagged wild type ERK3 (ERK3 WT) or ERK3 L290 mutants. As expected, ERK3 overexpression greatly increased HeLa cell migration (ERK3 WT versus empty vector (EV), Fig. 1a and b). Interestingly, as compared to ERK3 WT, both L290P and L290V mutants further significantly increased HeLa cell migration (Fig. 1b). We also determined the effects of L290 mutations on cell proliferation. Overexpression of ERK3 WT slightly, but not significantly, decreased HeLa cell proliferation, and there was no significant difference between ERK3 WT and either L290P or V mutants in their effects on cell proliferation (Fig. 1c).

**Figure 2:**
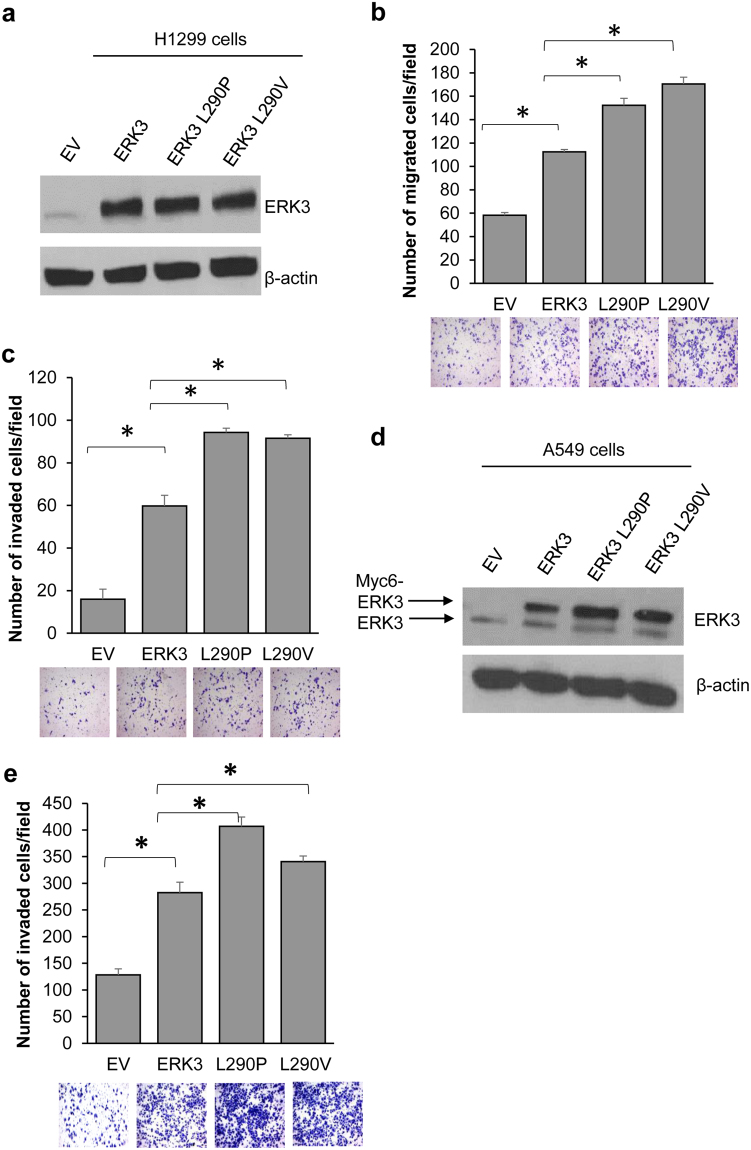
Both ERK3 L290P and L290V mutants have increased ability to promote lung cancer cell migration and invasion. (**a**) Western blot analysis of ERK3 and ERK3 mutants’ expression in H1299 cells transfected with an empty vector (EV), ERK3 wild type (ERK3), ERK3 L290P or ERK3 L290V plasmids as indicated. β-actin was used as a loading control. (**b**) The effect of L290P/V mutations on ERK3’s role in H1299 cell migration was analyzed by two-chamber transwell migration assay of H1299 cells transfected with an EV, wild type ERK3 or each of the ERK3 L290 mutants. Representative images are shown at the bottom. Quantitative results are presented as number of migrated cells per field. Values in bar graph represent mean ± S.D. of 3 separate experiments. *P < 0.05 by Student’s *t*-test. (**c**) Two-chamber transwell invasion assay of H1299 cells transfected with the indicated plasmids. Representative images are shown at the bottom. Quantitative results are presented as number of invaded cells per field. Values in bar graph represent mean ± S.D. of 3 separate experiments. *P < 0.05 by Student’s t-test. (**d**) Western blot analysis of ERK3 and ERK3 mutants’ expression in A549 cells transduced with lentiviral expression constructs as indicated. Please note that the lower bands in ERK3 blot are endogenous ERK3 proteins and the upper bands are exogenously expressed ERK3 or ERK3 L290P/V mutants that contain 6 Myc tags at the N-terminus. (**e**) Two-chamber transwell invasion assay of A549 cells with lentiviral transduction of EV, wild type ERK3 or each of the ERK3 L290 mutants. Representative images are shown at the bottom. Quantitative results are presented as number of invaded cells per field. Values in bar graph represent mean ± S.D. of 3 separate experiments. *P < 0.05 by Student’s *t*-test.

After revealing an important role for L290P/V mutations in promoting the migration of HeLa cells, we decided to confirm this finding in H1299 and A549 lung cancer cell lines that are directly relevant to the clinical finding of the presence of these mutations in lung cancer^2,16–18^. As shown in Fig. 2, as compared to ERK3 WT, both ERK3 L290P and L290V mutants have significantly increased capabilities of promoting migration and invasion of H1299 cells (Fig. 2a,b and c). As the efficiency of transient plasmid transfection in A549 cells is relatively poor, we exogenously overexpressed myc-tagged ERK3 WT or L290P/V mutants by lentiviral transduction. Similar to their effects in H1299 cells, both ERK3 L290P and L290V had significantly increased ability to promote the invasion of A549 cells as compared to that of ERK3 WT (Fig. 2d and e). Taken together, these results clearly demonstrate that both L290P and L290V mutations increase ERK3’s ability to promote cancer cell migration and invasion.

### L290P/V mutations do not affect ERK3 kinase activity

**Figure 3:**
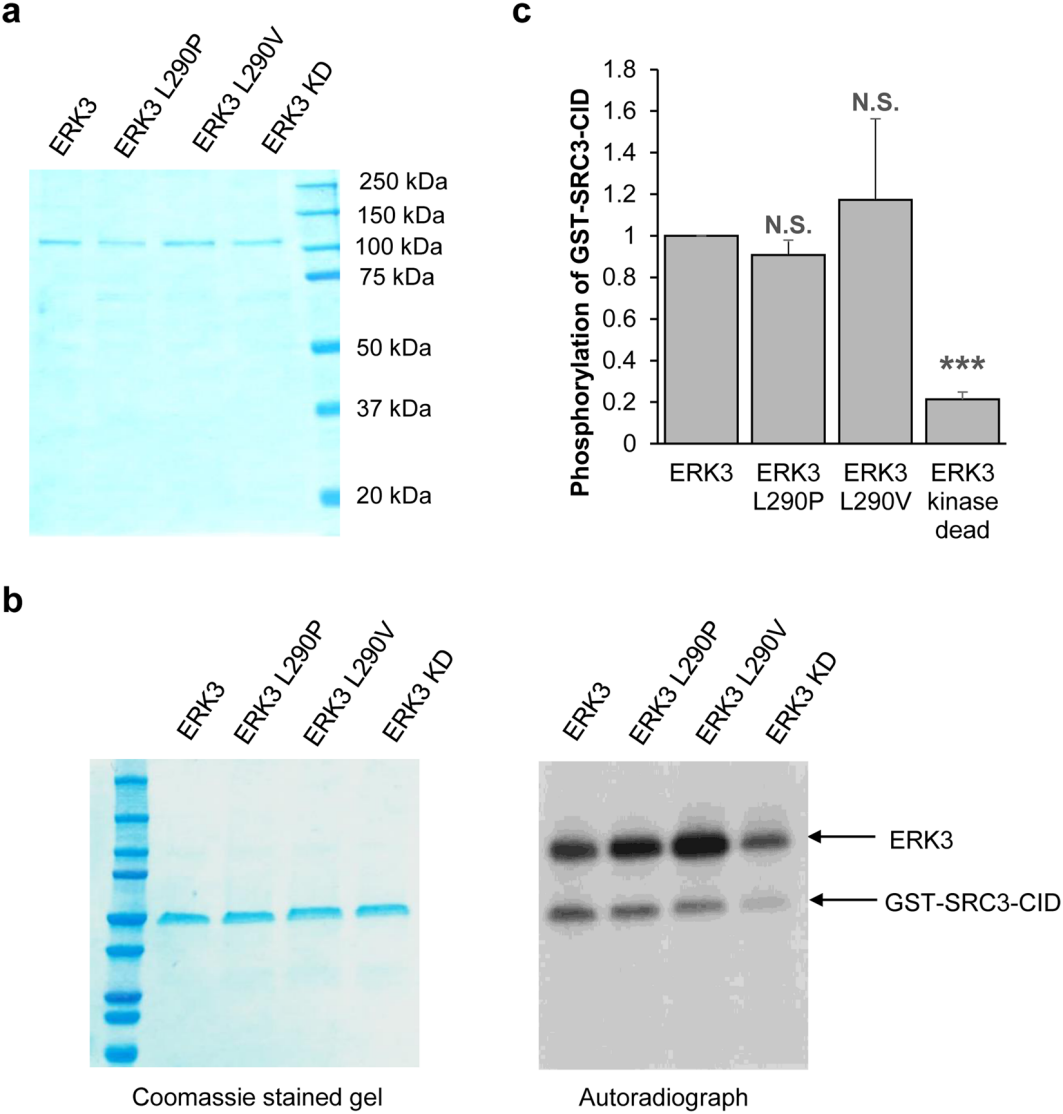
L290P/V mutations do not alter ERK3 kinase activity. (**a**) Coomassie staining of purified wild type or mutant ERK3 proteins. 293 T cells were transfected with HA-tagged wild type ERK3, ERK3 L290P, L290V or kinase dead (KD) plasmids. ERK3 proteins were immunoprecipitated using HA-antibody-conjugated agarose beads, followed by elution with HA peptide. The purified proteins (300 ng) were analyzed by SDS-PAGE gel followed by Coomassie staining. The molecular size of each protein marker is indicated on the right side. (**b**) *In vitro* ERK3 kinase assay was performed by incubating 100 ng of purified ERK3 or each of ERK3 mutants as indicated, together with 1 μg of recombinant GST-SRC3-CID (substrate) in the presence of γ-^32^P-ATP. Phosphorylation of GST-SRC3-CID by ERK3 proteins was detected by autoradiograph (the right panel). Total protein level of GST-SRC3-CID in the reactions is shown by Coomassie staining (the left panel). Please note that ERK3 proteins are hardly seen in the Coomassie-stained gel due to their small amount (100 ng). (**c**) Quantification of GST-SRC3-CID phosphorylation by wild type or mutant ERK3 proteins. The relative phosphorylation level of GST-SRC3-CID is represented by the ratio of the band intensity of phosphorylated GST-SRC3-CID (shown in the autoradiograph) over that of the corresponding total GST-SRC3-CID (shown in the commassie-stained gel). For the purpose of comparison, the nomalized phosphorylation level of GST-SRC3-CID by wild type ERK3 was arbitrarily set as 1.0. The bar graph represents the mean ± S.E. of 3 independent experiments. *P < 0.001 and N.S.: no significance by Student’s *t*-test.

To find out how L290P/V mutations increase ERK3’s ability to promote cancer cell migration and invasion, we first determined their effects on ERK3 kinase activity. ERK3 and each mutant cDNA were expressed in 293T cells, and ERK3 proteins were then purified using HA-antibody conjugated beads (Fig. 3a). The activities of purified ERK3 proteins were analyzed by *in vitro* kinase assay using the CBP-interacting domain of SRC-3 (SRC3-CID) as the substrate^7^. As expected, ERK3 kinase dead (ERK3 KD) has a remarkable reduction of kinase activity as demonstrated by the faint signal of SRC3-CID phosphorylation (Fig. 3b and c). Both ERK3 L290P and ERK3 L290V have similar kinase activity to that of wild type ERK3 (Fig. 3b and c).

**Figure 4:**
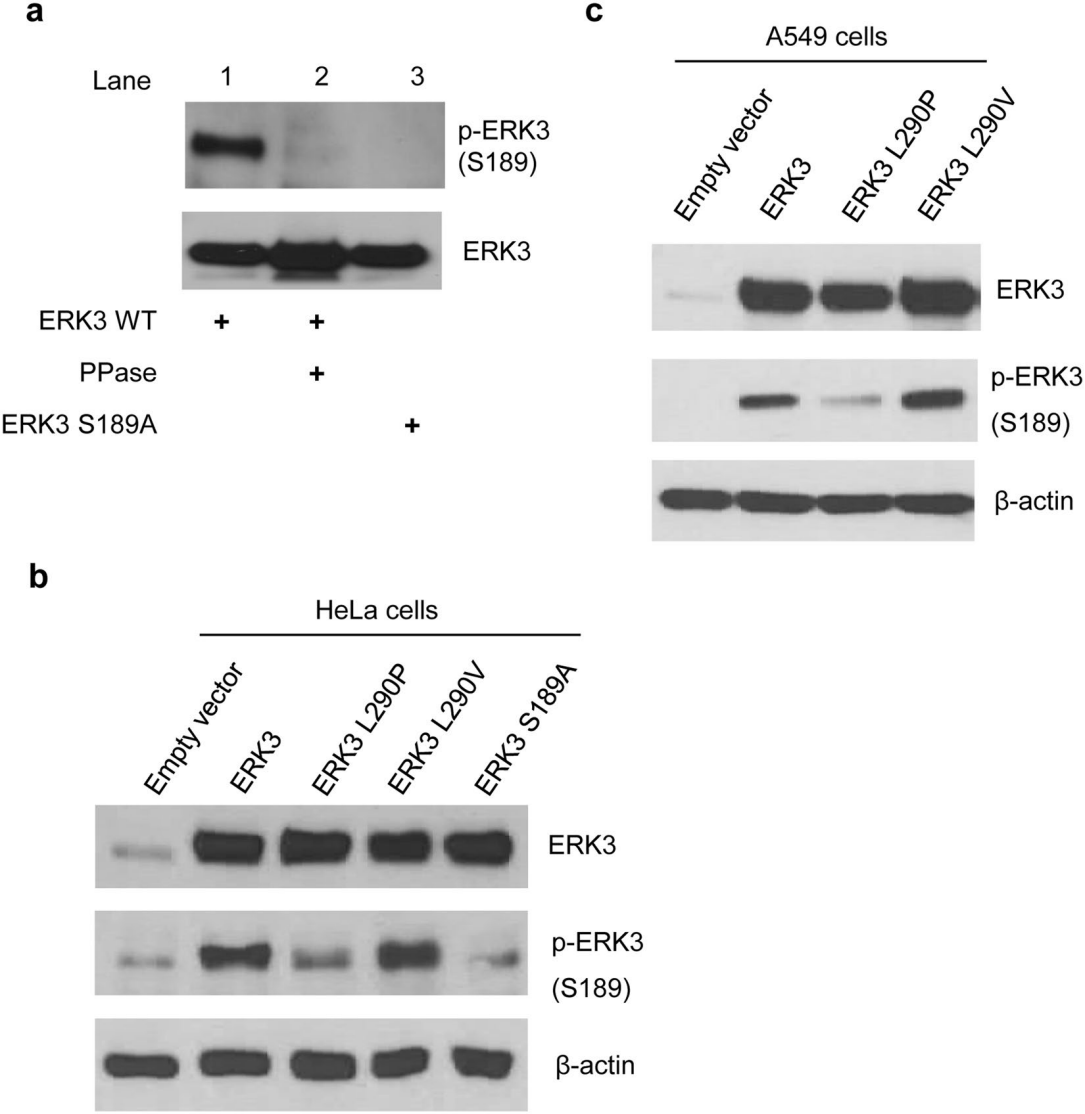
L290P mutation leads to a decrease in S189 phosphorylation of ERK3 protein, whereas L290V mutation has no clear effect. (**a**) Characterization of a phospho-S189 specific ERK3 antibody. 293 T cells were transfected with wild type ERK3 (ERK3 WT) or ERK3 S189A. Total cell lysates were treated with or without λ phosphatase (PPase). Phosphorylation of ERK3 at S189 [p-ERK3 (S189)] and expression level of ERK3 were determined using a phospho-S189 specific ERK3 antibody and an ERK3 antibody, respectively. (**b** and **c**) Western blot analysis of ERK3 phosphorylation at S189 in HeLa cells (**b**) and in A549 cells (**c**). Cells were transected with a pSG5 empty vector, HA-tagged wild-type ERK3, or each of the ERK3 mutants as indicated. Two days post-transfection, cells were lysed and levels of total ERK3 and ERK3 phosphorylated at S189 were analyzed by Western blotting. β-actin was used as a loading control.

We next tested whether these mutations alter the phosphorylation of S189 residue within the S-E-G activation motif, a supposed factor for enhancing ERK3 kinase activity. For this purpose, we generated a monoclonal antibody specifically against phosphorylated ERK3 at S189. As shown in Fig. 4a, phosphorylation of ERK3 WT protein was recognized by this antibody (Lane 1, phospho-ERK3 blot), and pre-treatment of ERK3 WT sample with λ-phosphatase abolished the phosphorylation signal (Lane 2, phospho-ERK3 blot). In addition, ERK3 S189A protein was not recognized by this phospho-S189 antibody (Lane 3, phospho-ERK3 blot). These results clearly demonstrate that this antibody specifically recognizes phosphorylated ERK3 at S189. We then determined the effects of L290P/V mutations on S189 phosphorylation of ERK3. As compared to wildtype ERK3, ERK3 L290P had greatly reduced S189 phosphorylation, whereas ERK3 L290V did not show clear change in both HeLa and A549 cells (Fig. 4b and c). Taken together, these results (in both Figs 3 and 4) suggest that the increased ability of L290P/V mutants to promote cancer cell migration and invasion is not owing to a change in kinase activity, although it is unclear as to why L290P shows no clear change in kinase activity but great reduction in S189 phosphorylation.

### ERK3 L290P/V mutants have increased cytoplasmic localization

**Figure 5:**
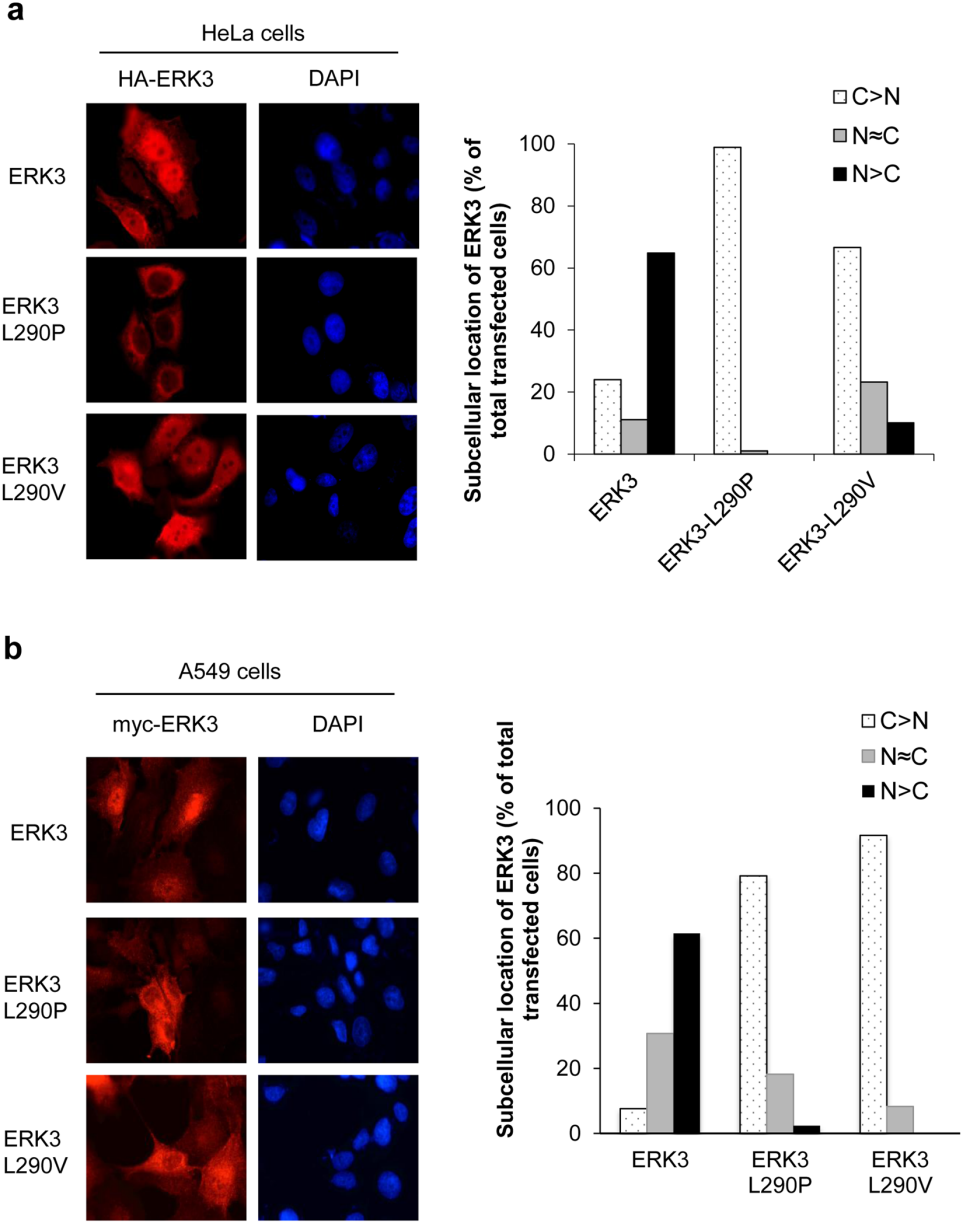
L290P/V mutations increase ERK3 protein’s cytoplasmic localization. (**a**) Subcellular distribution of wild type ERK3 and the L290P/V mutants in HeLa cells. HA-tagged wild type ERK3 or each of the L290P/V mutants was exogenously expressed in HeLa cells. Subcellular localization of ERK3 proteins was determined by immunofluorescent staining using an anti-HA antibody (red), and DNA was stained with DAPI (blue) to show the nucleus. Pictures were taken under 63X magnification and representative images are shown (left). For each transfection, at least 50 cells expressing ERK3 or each ERK3 mutant were analyzed and classified into three groups as follow: cells showing predominant cytoplasmic ERK3 localization (C > N), cells showing relatively equal distribution of ERK3 in the nucleus and cytoplasm (N = C) and cells showing predominant nuclear localization of ERK3 (N > C). The bar graph (right) represents the percentage of total transfected cells for each different group. (**b**) Subcellular distribution of wild type ERK3 and the ERK3 L290P/V mutants in A549 lung cancer cells. Experiments were done with the same procedures as described in (**a**), except that myc-tagged ERK3 was transduced into A549 cells by lentivirus and an anti-myc antibody was used for detecting the exogenously expressed ERK3 and ERK3 mutants.

ERK3 protein mainly localizes in the nucleus of cells, but it shuttles between the cytoplasm and nucleus. ERK3 nuclear export is mediated by CRM1^3^. Subcellular localization is known to regulate ERK3’s cellular functions^3^. We therefore tested whether L290P/V mutations alter ERK3’s subcellular localization, which may account for the increased ability in promoting cancer cell migration and invasion. As reported previously, ERK3 WT was mainly localized in the nucleus of HeLa cells (Fig. 5a). Strikingly, ERK3 L290P was seen to primarily localize in the cytoplasm. ERK3 L290V also has increased cytoplasmic localization (Fig. 5a). We confirmed the effects of L290P/V mutations on increasing the cytoplasmic localization of ERK3 in A549 lung cancer cells (Fig. 5b).

### ERK3 L290P/V mutants have increased interaction with CRM1

**Figure 6:**
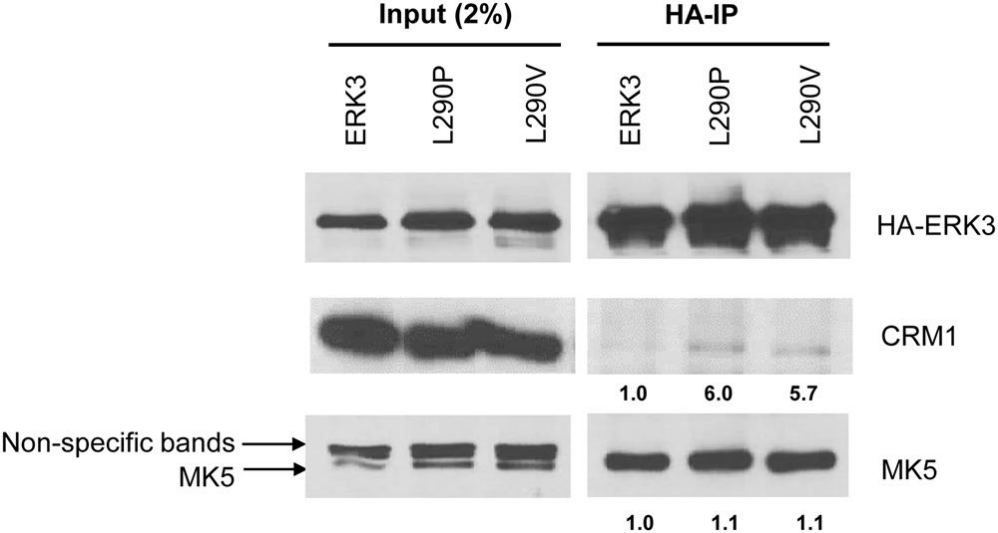
As compared to wild type ERK3, both L290P and L290V mutants have increased interactions with CRM1. HA-tagged wild type ERK3, ERK3 L290P or ERK3 L290V was exogenously expressed in HeLa cells. ERK3 protein complexes were immunoprecipitated using agarose beads conjugated with anti-HA antibodies, followed by Western blotting of the proteins as indicated in the figure. Input: 2% of the amount for immunoprecipitation (IP). Numbers below the immunoblots of CRM1 and MK5 in HA-IP samples represent the relative binding capacity of ERK3 (or L290P or V mutants) with these proteins, which is determined by the ratio of the band intensity in HA-IP over that in the corresponding input. For the purpose of comparison, the relative binding capacity of wild type ERK3 with either CRM1 or MK5 was arbitrarily set as 1.0.

To elucidate the molecular mechanism by which ERK3 L290P/V have an increased cytoplasmic localization, we tested the impact of these mutations on ERK3’s interaction with CRM1 and MK5, both of which are known to alter ERK3’s subcellular distribution. CRM1 mediates ERK3 nuclear export through direct binding^3^, and the interaction of ERK3 with MK5 leads to their co-transport to the cytoplasm from the nucleus^5,6^. We determined the interactions of ERK3 with CRM1 and MK5 by co-immunoprecipitation/Western blotting. While L290P/V mutations showed little effect on ERK3’s interaction with MK5, they both greatly increased ERK3’s interaction with CRM1 (Fig. 6), which may account for the increase in their cytoplasmic localization.

## Discussion

Accumulating evidence has demonstrated the important role of ERK3 in human cancers, such as promoting cancer cell migration and invasion and conferring cancer cells drug resistance^4,7,8,12,13^. In addition, ERK3 expression is upregulated in several cancers including non-small cell lung cancer and oral squamous cell carcinoma^7,10^. However, it is virtually unknown whether and how ERK3 kinase activity is altered in cancers. As protein kinases are frequently altered by mutations in cancers, we attempted to investigate cancer-related ERK3 mutations. By searching various literature^2,16–18^ and the COSMIC database, we found that leucine 290 within the kinase domain of ERK3 is mutated to either proline or valine in multiple cancer types, albeit at a low frequency. Importantly, our present findings show that as compared to the wild type ERK3, both L290P and L290V mutants have greatly increased ability to promote cancer cell migration and invasion. This is unlikely to be due to a change in kinase activity as neither L290P nor L290V mutations significantly altered ERK3 kinase activity. Interestingly, both mutants show an increase in interaction with CRM1 and in cytoplasmic localization, which may contribute to the increased ability in promoting cell migration and invasion. Of note, ERK3 was shown to interact with Group I p21-activated kinases (PAKs) that mainly localize in the cytoplasm and are critical regulators of cytoskeletal structure and cell motility^19,20^. Thus, it would be interesting to investigate whether ERK3 and L290P/V mutants are involved in PAKs-mediated cell migration process when they are localized in the cytoplasm.

ERK3 protein is mainly localized in the nucleus of cultured cells, but can be exported to the cytoplasm and even be localized to the leading edge of the plasma membrane^3,4^. However, no functional nuclear export signal (NES) has been identified for ERK3 although the region from amino acids 297–542 was shown to be important for nuclear export^3^. The region surrounding L290 does not appear to be a classical leucine-rich NES. ERK3 nuclear export is shown to be actively mediated by CRM1^3^. Indeed, both L290P and L290V mutations confer ERK3 increased interaction with CRM1. While L290P/V mutants have increased cytoplasmic localization, their kinase activities show no clear change in comparison with that of wild type ERK3. Intriguingly, ERK3 L290P mutant has a remarkable reduction in S189 phosphorylation, whereas ERK3 L290V shows little change on this. These results are actually consistent with the previous finding that nuclear export of ERK3 is not dependent on its kinase activity or S189 phosphorylation^3^.

ERK3 promotes both migration and invasion of cancer cells. While ERK3 is known to phosphorylate SRC-3 and upregulate SRC-3-mediated expression of pro-invasive MMPs, how ERK3 regulates cell morphology and motility remains elusive. ERK3 interacts with MK5 and stimulates MK5’s kinase activity toward HSP27^5,19–21^. In addition, ERK3 is capable of co-transporting MK5 from the nucleus to the cytoplasm. While MK5/HSP27 axis was convincingly shown to promote actin rearrangement, inconsistent findings were reported about its role in motility, some showing a promoting role^22,23^ and others indicating an inhibitory effect^24,25^. It remains to be explored whether or not MK5 is a downstream effector of ERK3 in promoting cell migration. Nevertheless, L290P/V mutations do not seem to affect ERK3’s interaction with MK5. Another intriguing aspect about ERK3’s role in cell migration is whether or not ERK3 kinase activity is involved in this process. Surprisingly, the effect of ERK3 on MDA-MB-231 breast cancer cell spreading and elongation appeared to be independent of kinase activity^4^, indicating that ERK3 may have a kinase-independent role in cell motility. Of note, L290P/V mutants do not exhibit changes in kinase activity although they have a greatly increased ability in promoting cancer migration. Although the molecular mechanisms underlying ERK3’s role in promoting cell motility remain to be further elucidated, our current findings suggest that it might be associated with ERK3’s localization to the cytoplasm, where ERK3 targets some factors directly involved in cell motility.

In summary, our study demonstrates that ERK3 L290P/V mutants display increased cytoplasmic localization and capability in promoting cancer cell migration and invasion. It not only provides better understanding on the compartmental control of ERK3’s cellular functions, but also reveals a new mechanism by which ERK3 promotes cancer cell invasiveness.

## Methods

### Cell culture and transfection

HeLa and 293T cells were cultured in Dulbecco’s Modified Eagle Medium (DMEM) supplemented with 10% fetal bovine serum (FBS) and antibiotics. H1299 and A549 lung cancer cells were maintained in RPMI-1640 medium supplemented with 10% FBS and antibiotics. All the culture media and supplements were purchased from Gibco, Thermofisher Scientific. Based on the information in COSMIC database, cell lines tested in the current study, including A549, H1299 and HeLa, do not harbor any mutation on ERK3. Plasmid transfections were done using Lipofectamine 3000 (Invitrogen, Thermofisher Scientific) according to the manufacturer’s instructions.

### Expression plasmids

The mammalian expression plasmid of ERK3 with HA tag at the N-terminus (pSG5-HA-ERK3) was used for generating mutant plasmids by site-directed mutagenesis using the QuickChange II Site-Directed Mutagenesis Kit (Agilent Technologies). The following mutant plasmids were generated: two leucine 290 mutants (ERK3 L290P and L290V), a kinase dead (KD) mutant in which K49/50 in the ATP-binding site of ERK3 were mutated to alanine, as well as an S189A mutant in which the S189 phospho-acceptor site in the S-E-G activation loop of ERK3 was mutated to alanine. The primers used for mutagenesis are listed in Table 1. All ERK3 mutant plasmids generated were verified by sequencing.

**Table 1:** List of primers used for site-directed mutagenesis.

Mutant plasmids	Primers used for site-directed mutagenesis
ERK3 L290P	5′-aatttgttccaggaaatccggtgcttctcgactaattcc-3′
ERK3 L290V	5′-tccaggaaatccactgcttctcgactaattcctgg-3′
ERK3 S189A	5′-cccataagggtcatcttgctgaaggattggttac-3′
ERK3 kinase dead (KD)	5′-ggggatcagtaaggacaattgccgcgatggctactcttttgtcac-3′

For lentiviral transduction of ERK3, the previously described pCDH-Myc6-ERK3 was used^7^. pCDH-CMV-MCS-EF1-Puro was used as an empty vector control. pCDH-Myc6-ERK3 L290P and L290V mutants were generated by inserting the fragments released from pSG5-HA-ERK3 L290P or L290V plasmids by ApaI/XhoI digestion into pCDH-Myc6-ERK3 digested with the same restriction digestion enzymes. The plasmid used for bacterial expression of GST-SRC-3 CID (aa 841–1080) was described previously^26^.

### Lentiviral transduction

Pseudotyped lentiviruses were produced in 293T cells by co-transfecting lentiviral expression constructs and Trans-Lentiviral Packaging Plasmid Mix following the manufacturer’s instructions (Open Biosystems). 48 hours post transfection, the pseudoviral particles were harvested and concentrated using PEG-it Virus Precipitation Solution (System Biosciences). A549 cells were transduced with prepared lentivirus in the presence of polybrene (4 μg/ml) for two days.

### Western blotting

Cell lysis and western blotting was done as described previously^7,12^. The following primary antibodies were used in Western blotting: anti-ERK3 (Abcam, Cat# ab53277), anti-p-ERK3 (S189) (generated for our lab by the monoclonal antibody core facility at Baylor School of Medicine, using YSHKGHL(pS)EGLV phsophopeptide sequence as an antigen), anti–β-actin (Sigma-Aldrich, Cat# A5316), anti-CRM1 (Santa Cruz Biotechnology, Cat# sc-74454) and anti-MK5 (Cell signaling, Cat# D70A10).

### Two-chamber transwell cell migration and invasion assay

Cell migration and invasion were analyzed using a modified 2-chamber transwell system (BD Biosciences) as described previously^7^. Cancer cells suspended in serum-free medium were added to transwell inserts, while culture media supplemented with 10% FBS was added to each bottom well. Cells were added in each transwell insert and allowed to migrate for 19 hours (for H1299 cells) or 20 hours (for HeLa cells) in a 37 °C cell incubator. Cells in the upper surface of the transwell were removed using cotton swabs. Migrated cells attached on the undersurface were fixed with 4% paraformaldehyde for 15 minutes and stained with crystal violet solution (0.5% in water) for 10 minutes. Migrated cells were then photographed and counted under a microscope at 50X magnification.

The cell invasion assay was performed by following the same procedures as those for the cell migration assay, except that the transwell inserts were pre-coated with Growth Factor-Reduced Matrigel (BD Biosciences) and the cells were allowed to invade during 20 hours.

### Cell proliferation assay

Cell proliferation was determined using the CellTiter 96 AQueous One Solution Cell Proliferation Assay Kit (Promega) following the manufacturer’s instructions.

### Protein purification

HA-tagged ERK3 WT or mutant proteins were purified from mammalian cells as described previously^7^. pSG5-HA-ERK3 WT, L290P, L290V or KD were transfected in 293T cells. Two days post-transfection, cells were lysed and the protein lysate supernatant was incubated with anti-HA affinity agarose beads (Sigma-Aldrich, Cat# E6779). After washing the beads, the proteins were eluted using HA peptide (Sigma-Aldrich). The protein concentrations were determined by bradford protein assay, and protein purity was assessed by running 300 ng purified proteins on a 10% SDS-PAGE gel followed by staining with coomassie blue solution (Expedeon). GST-SRC3-CID (aa 841–1080) was expressed in *E. coli* and purified using Glutathione Sepharose 4B (GE Healthcare Life Sciences) following the manufacturer’s instructions.

### *In vitro* ERK3 kinase assay

The *in vitro* kinase assay was carried out as described previously^7,8^. Each reaction contained 100 ng of purified ERK3 proteins and 1 μg of the purified protein substrate GST-SRC3-CID, 5 μCi γ-^32^P-ATP (Perkin Elmer) and 25 μM cold ATP. The reaction was carried out at 30 °C for 30 minutes and then stopped by SDS sample buffer and boiling. Proteins were resolved by SDS-PAGE gel, stained with coomassie blue stain and visualized by autoradiography. Densitometry analysis of the bands was done by IMAGEJ software.

### Immunofluorescence

HeLa cells were transfected with pSG5-HA-ERK3 WT or L290P or L290V plasmids, while A549 cells were transduced with lentiviral vectors encoding pCDH-Myc6-ERK3 WT or L290 mutants. Two days later, exogenously expressed ERK3 proteins were immuno-labelled with anti-HA antibody (Sigma-Aldrich, Cat# H3663) or anti-myc antibody (Cell Signaling, Cat# 2276) appropriately following the procedures described previously^27^. This was followed by the fluorescent labeling with Alexa Fluor secondary antibody (Invitrogen, Cat# A11032) and DAPI staining of cell nuclei. Images were captured with a Leica CTR 6000 Microscope (Leica Microsystems) and analyzed using IMAGEPRO 6.2 software (Media Cybernetics).

### Co-immunoprecipitation

HeLa cells were transfected with pSG5-ERK3 WT, L290P or L290V plasmids. The cells were lysed two days post-transfection as described previously^7^. The protein lysate supernatant was precleared using Protein A Affinity gel beads then HA-tagged ERK3 proteins were immunoprecipitated using anti-HA affinity agarose beads (Sigma-Aldrich Cat# E6779). The beads were washed then the proteins were boiled off the beads in SDS sample buffer, resolved on 8% SDS-PAGE gels and Western blotting done as described before. 2% of the amount of protein supernatant for immunoprecipitation was loaded as the input control.

### Statistics

Results are expressed as mean ± standard deviation (S.D.) or standard error (S.E.) as indicated in each figure legend. Statistical significance was determined by a 2-tailed Student’s *t*-test. A *P* value of less than 0.05 was considered statistically significant.

### Data availability

All data generated and analysed during this study are included in this published article
